# Role of protein phosphatases PP1, PP2A, PP4 and Cdc14 in the DNA damage response

**DOI:** 10.15698/cst2019.03.178

**Published:** 2019-02-21

**Authors:** Facundo Ramos, María Teresa Villoria, Esmeralda Alonso-Rodríguez, Andrés Clemente-Blanco

**Affiliations:** 1 Cell Cycle and Genome Stability Group. Institute of Functional Biology and Genomics (IBFG). Spanish National Research Council (CSIC), University of Salamanca (USAL), C/Zacarías González 2, Salamanca 37007, SPAIN.

**Keywords:** phosphatases, DNA damage response, PP1, PP2A, PP4, Cdc14

## Abstract

Maintenance of genome integrity is fundamental for cellular physiology. Our hereditary information encoded in the DNA is intrinsically susceptible to suffer variations, mostly due to the constant presence of endogenous and environmental genotoxic stresses. Genomic insults must be repaired to avoid loss or inappropriate transmission of the genetic information, a situation that could lead to the appearance of developmental anomalies and tumorigenesis. To safeguard our genome, cells have evolved a series of mechanisms collectively known as the DNA damage response (DDR). This surveillance system regulates multiple features of the cellular response, including the detection of the lesion, a transient cell cycle arrest and the restoration of the broken DNA molecule. While the role of multiple kinases in the DDR has been well documented over the last years, the intricate roles of protein dephosphorylation have only recently begun to be addressed. In this review, we have compiled recent information about the function of protein phosphatases PP1, PP2A, PP4 and Cdc14 in the DDR, focusing mainly on their capacity to regulate the DNA damage checkpoint and the repair mechanism encompassed in the restoration of a DNA lesion.

## INTRODUCTION

Cells are constantly suffering endogenous and exogenous stresses that affect the integrity of the genetic material. It has been estimated that every cell of our body is exposed to about 10^5^ lesions per day. In response to such levels of DNA damage, cells have evolved sophisticated mechanisms that safeguard the stability of our genome. These mechanisms, collectively enclosed under the name of DNA damage response (DDR), are constantly surveying the genome to detect DNA errors and fix them [1, 2]. When these mechanisms fail or the rate of DNA damage exceeds the capacity of the cell to deal with it, the increased accumulation of genetic alterations can overwhelm the cell resulting in the appearance of a malignant transformation. As a matter of fact, multiple congenital human disorders have been directly linked to a failure in executing this surveillance pathway, mirroring the importance of the DDR in the maintenance of genome integrity for health and development in humans [3-6].

During the last years, there have been rapid progresses in the characterization of the mechanisms governed by the DNA damage response. Upon generation of a DNA lesion, the main function of the DDR is to couple cell cycle with DNA repair. This is attained by triggering two inter-connected pathways: 1) the DNA damage checkpoint, a molecular mechanism that restrains cell cycle progression to avoid the segregation of the duplicated chromosomes until the broken DNA has been restored, and 2) the activation of specific repair factors responsible for the correct execution of the different phases encompassed in the repair process. The correct activation and coordination of both routes by the DDR ensures a proficient and timely restoration of the DNA molecule. Today we know that the transmission of the signal along these pathways is mainly driven by phosphorylation events by specific kinases that phosphorylate DDR components predominantly at serine and threonine residues [7-9]. However, less is known about the role of protein dephosphorylation by protein phosphatases and their implication in the restoration of a DNA lesion. Still, it is reasonable to think that the fine-tuning of the response relies on the activity of phosphatases in order to prevent illegitimate activation of the DDR in the absence of damage as well as to allow a rapid cessation of the signal once the DNA lesion has been fixed. Because of this perspective, most of the studies involving protein phosphatases have focused on their role in counterbalancing DDR-kinases to stimulate cell cycle re-entry upon repair. However, in the last years several studies have revealed that these enzymes are also able to directly modulate the DDR at the repair level, a discovery that has changed the perception of protein dephosphorylation in the response to DNA damage.

To date, four different phosphatases have been mainly involved in the DDR: the Ser/Thr protein phosphatase-1 (PP1), the protein phosphatase 2A (PP2A), the protein phosphatase 4 (PP4) and the Cdk-antagonizing phosphatase CDC14. These phosphatases can be classified into two groups on the basis of their sequence, structure and biological activity. PP1, PP4 and PP2A are comprised in the classic Ser/Thr phosphoprotein phosphatases (PPPs) family while Cdc14 forms part of the dual-specificity phosphatase (DUSP) family. One peculiarity of these phosphatases is their ability to counteract a great number of kinases. It has been estimated in human cells that there are about 500 protein kinases, while only 150 phosphatases have been described up to date [10, 11]. Due to this difference between the number of kinases and phosphatases, it has always been considered that phosphatases are promiscuous enzymes. Today we know that these proteins are indeed selective and tightly regulated enzymes. The discrimination in target recognition by protein phosphatases is attained by their ability to form specific complexes between a catalytic subunit and multiple regulatory elements that confer the specificity to the holoenzyme [12-14]. Each of these multimeric holoenzymes works as a distinct signalling entity by modulating the activity of the catalytic subunit and creating their own substrate specificity. Actually, taking into account the great number of phosphatase complexe rearrangements identified *in vivo* recent studies postulated that protein phosphatases exhibit similar complexity and specificity as protein kinases.

It is important to note that regulation of protein phosphorylation/dephosphorylation during the DDR is critical to maintain genome integrity and prevent the development of diseases such as cancer. Phosphatases are involved in the control of DDR activation after a DNA lesion is generated, as well as to its inactivation when the DNA adduct has been repaired. It is generally accepted that this control might be hijacked by cancer cells to elude the activation of checkpoint pathways during tumorigenesis, allowing tumor cells to grow uncontrolled. Supporting this notion, several types of cancer show an altered regulation of the DDR, a fact that may explain the accumulation of high levels of DNA damage at later stages of the disease. In addition, most oncogenes encode for protein kinases and phosphatases, reflecting the importance of protein phosphorylation in cancer development and progression. Interestingly, protein phosphatases can also operate as tumor suppressors through positive regulation of the DDR [13, 15]. In this regard, these enzymes have been implicated not only in the control of the DNA damage checkpoint, but also in the regulation of the repair mechanisms operating in the response. Thus, even though it is quite premature to consider protein phosphatases as specific targets to tackle cancer progression, it is nevertheless an attractive field to work on.

**Figure 1:**
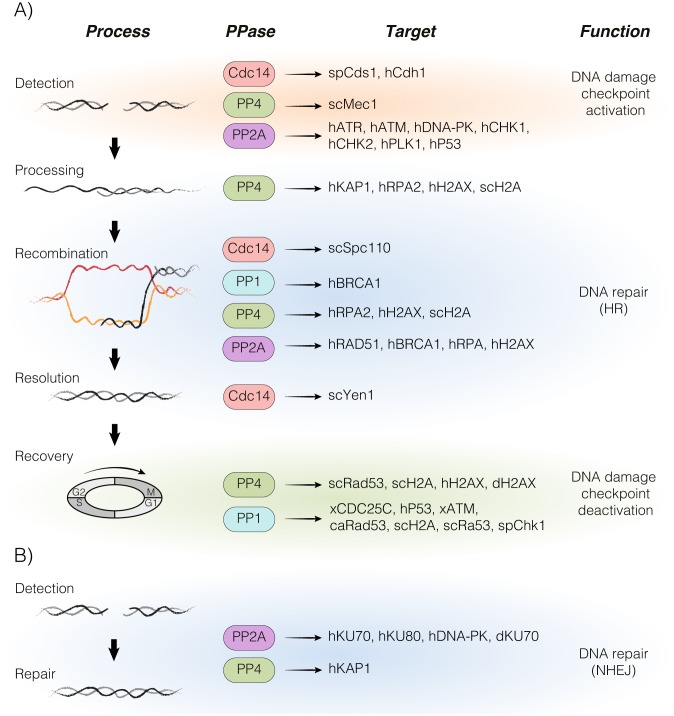
FIGURE 1: A global overview of the protein dephosphorylation landscape in the DDR. The figure summarizes the participation of PP1, PP2A, PP4 and Cdc14 in each step of the DNA damage response. All phosphatase's targets identified in different model organisms are also depicted (sc, *Saccharomyces cerevisae*; sp, *Schizosaccharomyces pombe*; ca, *Candida albicans*; d, *Drosophila melanogaster*; x, Xenopus; h, Human). The involvement of each protein phosphatase in the homologous recombination **(A)** and non-homologous end joining **(B)** pathways is portrayed. HR – homologous recombination, NHEJ – non-homologous end joining.

In this review, we summarize recent advances in the fundamental principles behind the main DDR-phosphatases PP1, PP4, PP2A and CDC14 in the repair of a DNA lesion and their physiological significance in the regulation of the DNA damage response (**Figure 1**). We also discuss the potential role of these phosphatases in cancer progression and treatment.

## PP1: FROM CELL CYCLE REGULATOR TO A KEYSTONE MEMBER OF THE DNA DAMAGE RESPONSE

Among all Ser/Thr phosphatases, protein phosphatase-1 (PP1) is perhaps the most broadly and best studied. PP1 is responsible for the majority of the dephosphorylation events taking place in the cell. It has been estimated that one third of all phosphoproteins are dephosphorylated by this phosphatase [16]. As other phosphatase complexes, PP1 is formed by a catalytic subunit and multiple regulatory elements that provide specificity for multiple targets. The central region of the catalytic subunit is practically identical in amino acid sequence between different species, while specific species-dependent variations are observed in the N- and C-terminal domains. Interestingly, the conserved central domain of PP1's catalytic subunit is shared with PP2A, a feature that could explain the redundancy in protein functions observed between both complexes. As mentioned above, a hallmark of the PP1 enzyme is that its catalytic subunit always works in complex with regulatory elements. Most PP1 regulatory elements interact with the catalytic subunit through a conserved binding region known as the RVxF motif [17]. Up to date, there have been identified more than 200 PP1's interacting proteins, a feature that reflects the vast number of cellular functions attributed to this holoenzyme including glucose metabolism, transcription, cytoskeleton organization, cell cycle and meiosis [18].

One of the first observations that underlined a possible role for PP1 in controlling checkpoints activity was the discovery that its overexpression bypassed the spindle assemble checkpoint (SAC) arrest triggered during the response to spindle-unattached chromosomes [19]. Sister chromatids without tension generate a signal that is transmitted by the Ipl1 and Mps1 kinases to stabilize the separase inhibitor Pds1, thus restraining nuclear segregation [20]. At present, we know that SAC inactivation by PP1 is carried out by its ability to dephosphorylate multiple Ipl1's substrates [21]. A similar role was also found for PP1 in the control of meiosis. Red1 phosphorylation by Mek1 activates the pachytene checkpoint in response to defects in meiotic recombination and/or chromosome synapsis. However, when meiotic recombination has been accomplished, Red1 dephosphorylation by PP1 relieves the checkpoint arrest to stimulate cell cycle progression [22].

In the last years, several lines of investigation have involved PP1 in the DNA damage checkpoint by directly modulating the steady state phosphorylation of DDR factors. One of the first evidences involving PP1 in the DNA damage response came from a screening in *Schizosaccharomyces pombe* to isolate genes that, when overexpressed, resulted in premature mitotic entry in the presence of genotoxic stress. In this screening, Dis2 (main subunit of the PP1 complex in the fission yeast) was identified as the only requirement to endorse cell cycle re-entry upon DNA repair by dephosphorylating the DNA damage checkpoint effector Chk1 [23]. Interestingly, PP1 was not required for cell cycle resumption in response to replication stress, suggesting that the role of the phosphatase in the control of the DDR in the fission yeast was restricted to enhance cell recovery from G2/M arrested cells responding to physical DNA lesions [23]. In *Saccharomyces cerevisiae*, PP1 is required for the dephosphorylation of histone γ-H2A and Rad53, a prerequisite for cell cycle resumption from replication stress, extending its role in DDR silencing to the budding yeast [24]. Excitingly, the role of PP1 in checkpoint deactivation must be regulated by its interaction with regulatory elements since Sds22, a well-known regulatory subunit of PP1 in the fungal pathogen *Candida albicans*, mediates Rad53 dephosphorylation in response to alkylating agents [25].

It has been well documented that the DNA damage checkpoint silencing function of PP1 is evolutionary conserved in higher eukaryotes. PP1 is responsible for controlling the threshold of DNA damage checkpoint activation by opposing ATM activity in Xenopus [26]. Importantly, PP1-dependent regulation of ATM is mediated by its regulatory subunit Repo-Man, a factor that stimulates the binding of the holoenzyme to chromatin [26]. The role of PP1 in controlling the levels of checkpoint activation has also been extended to human cells, since it has been demonstrated that inhibition of the damage-dependent p53 signalling pathway depends on PP1 activity [27, 28]. Importantly, p53 attenuation is attained by its direct dephosphorylation at Ser15 and Ser37 [29] in a process mediated by different PP1 regulatory factors such as PNUTS [30], p53BP2 [31] and GADD34 [28]. Another well-known substrate of PP1 during the DNA damage checkpoint in higher eukaryotes is the mitotic phosphatase CDC25. CDC25 stimulates mitotic entry by removing inhibitory phosphorylation on the CDK1 subunit Cyclin B [32]. In addition, human CDC25C is phosphorylated by CHK1 and CHK2 in response to DNA damage to prevent its transport into the nucleus, thus restraining mitotic entry [33]. Taking into account that PP1 dephosphorylates CDC25C during the G2/M transition in Xenopus, it is tempting to speculate that cell cycle re-entry might be enhanced by the positive effect that PP1 exerts over CDC25C. However, as CDC25C has been shown to be dispensable for DNA damage checkpoint activation in human and mouse cells [34, 35], the molecular significance of PP1-dependent CDC25C dephosphorylation remains controversial.

Even though the principal function of PP1 in the DDR seems to be cell cycle re-entry upon repair, it has recently been postulated that PP1 could have also a role in the direct modulation of the repair machinery. In this line, human PP1 binds and dephosphorylates hCds1/Chk2-phosphorylated BRCA1, a function that is essential for its role in promoting recombinational DNA repair following γ-radiation exposure [36, 37]. Moreover, depletion of PP1 affects NHEJ (Non-homologous end joining) in both Xenopus and humans [38], establishing a direct role of the phosphatase in the physical restoration of double-strand breaks (DSBs). Importantly, the regulation of PP1 activity by its regulatory subunits along the DDR is also important for the correct execution of the repair process, as the expression of a covalent fusion of PP1 with the regulatory element NIPP1 results in the generation of RNA-DNA hybrids (R-loops), enhanced chromatin compaction, slow replication fork progression and a reduced capacity to deal with DNA lesions [39].

## PP2A: AN ESSENTIAL FACTOR FOR THE REGULATION OF DNA DAMAGE CHECKPOINT AND DNA REPAIR

PP2A is a Ser/Thr protein phosphatase belonging to the PPP family of phosphatases conserved in higher eukaryotes. It operates as a heterotrimeric complex consisting of a catalytic subunit (PPP2Cα and PPP2Cβ), a scaffolding subunit (Aα and Aβ), and multiple regulatory subunits (B). The large number of regulatory elements allows the formation of numerous functionally distinct PP2A complexes, explaining the vast number of cellular functions attributed to PP2A [40-42]. It is believed that these regulatory subunits dictate the subcellular localization of the holoenzyme and confer substrate specificity. In the eukaryotic model *S. cerevisiae*, PP2AC is encoded by two identical genes, *PPH21* and *PPH22*, each of them contributing to approximately half of the PP2A activity in the cell [43, 44]. *TPD3* codifies the subunit A [45] and the regulatory subunits are encoded only by two known distinct genes, *CDC55* and *RTS1* [46, 47].

PP2A is one of the most well-studied phosphatases and has been implicated in the regulation of many cellular processes including cell cycle progression [48, 49], DNA replication, gene transcription/translation [40], cell differentiation [50] and DNA damage response [51]. Of all these functions, probably the best characterized is the regulation of the G2/M transition. PP2A involvement in cell cycle regulation was originally suggested by several findings showing that its inactivation promoted premature mitotic entry in fission yeast [52]. This observation was also reproduced in budding yeast experiments demonstrating that elimination of the PP2A regulatory subunit Cdc55 resulted in a similar premature mitotic entry due to the loss of function of the holoenzyme [53]. In Xenopus, PP2A regulates the G2/M transition by modulating the phosphorylation levels of the mitotic phosphatase Cdc25 [54], while in *S. cerevisiae* it seems that its main effector is the kinase Swe1 [55]. A similar molecular mechanism has also been postulated in *S. pombe* [52] and humans [56]. Importantly, Cdc25 and Swe1 are not the only targets of the phosphatase during the G2/M transition. It has also been reported in *S. cerevisiae* that PP2A acts as a negative regulator of sister chromatids separation by counteracting polo-kinase phosphorylation of Scc1 to inhibit cohesin cleavage [57]. A similar mechanism has been described in centromeric cohesion during mitosis in human cells [58].

The awareness of a role of PP2A in the DDR came from the observation that elimination of its function by treating mice with okadaic acid in a two-stage carcinogenesis experiment led to an increase in tumorigenesis [59, 60]. Today we know that PP2A impacts on the damage response by regulating the activity of the primary (ATM, ATR and DNA-PK) and secondary (CHK1 and CHK2) kinases involved in the signalling cascade. It has been reported that PP2A-deficient cells display an increased level of ATM auto-phosphorylation/activation accompanied by an upregulation of the ATM downstream kinase CHK2 [61, 62]. This results in the activation of a G1/S arrest and the down-regulation of RAD51 and BRCA1, which mediate DNA repair by homologous recombination (HR) [62]. Consistent with a general role of PP2A in the DDR, several studies have reported that PP2A dephosphorylates not only ATM and CHK2 but also the ATR kinase and its downstream target CHK1. Surprisingly, this function of PP2A is directly involved in the maintenance of a low checkpoint activation in an unperturbed cell cycle while allowing a rapid release from this regulation immediately after DNA damage induction [63-65]. A similar hypothesis has been postulated in endothelial cells in response to oxidative stress. In this regard, PP2A induces a rapid dephosphorylation of the protein nucleophosmin, which is translocated from the cytoplasm into the nucleus, thus preventing the formation of γ-H2AX foci [66]. Furthermore, the role of PP2A in controlling the DNA damage checkpoint is reinforced by its ability to dephosphorylate p53. PP2A binds and dephosphorylates p53 at Ser37 after DNA damage, thereby controlling its transcriptional activity [67]. Moreover, dephosphorylation of p53 at Thr55 upon DNA damage stabilizes the protein to enhance a proficient DNA damage checkpoint activation [68]. It is important to remark that PP2A-dependent DDR inhibition is also operating to integrate metabolic signals within the response. In cells experiencing replication stress, PP2A/PP2A-like acts in a network with Irc21 (cytochrome b5 reductase implicated in the production of ceramide) and TORC1 to attenuate the ATR cascade [69]. Once the DNA damage checkpoint has been activated, PP2A also cooperates in its maintenance by dephosphorylating and inhibiting Polo-like kinase 1 (Plk1), a positive regulator of the G2/M transition, thus stimulating a G2 arrest [70-72].

Excitingly, PP2A is not only required for DNA damage checkpoint regulation, but also for DNA repair. It is already known that in response to DNA damage induced by hydroxyurea (HU), the single-stranded (ss)DNA-binding protein RPA32 is phosphorylated at Thr21 and Ser33 in an ATM/ATR-dependent manner. It is believed that RPA32 phosphorylation suppresses DNA replication and enhances the recruitment of other checkpoint/repair proteins to DNA lesions. Interestingly, PP2A-mediated RPA32 dephosphorylation is required for the completion of the DNA repair process [73]. This result is reinforced by the observation that PP2A is involved in regulation of the steady-state phosphorylation of H2AX. Indeed, it has been demonstrated that dephosphorylation of γ-H2AX during DNA repair removes it from nuclear foci near the break sites and elicits DNA repair. PP2A-dependent γ-H2AX removal from chromatin is directly linked to its role in enhancing DNA repair as PP2A-deficient cells are affected in the restoration of a DNA lesion and are hypersensitive to DNA-damaging agents [74, 75]. Modulation of RPA and H2AX phosphorylation levels directly connects this phosphatase with the accomplishment of the HR pathway. However, PP2A has also been involved in stimulating NHEJ since it dephosphorylates DNA-PKcs, Ku70 and Ku80, *in vitro* [76]. Similar data were also obtained in *Drosophila melanogaster*, where failure of the B55-mediated dephosphorylation generates abnormal and/or untimely phosphorylation of Ku70, which interferes with DNA repair and causes the appearance of chromosome aberrations [77].

## PP4: A PHOSPHATASE COMPLEX WITH MULTIPLE ROLES IN THE DNA DAMAGE RESPONSE

Over two decades since its discovery in the nineties, protein phosphatase 4 (Ppp4/PP4/PPX) has earned a recognised place as a ubiquitous Ser/Thr phosphatase that regulates many cellular functions independently of other protein phosphatases of the PPP family. Mammalian Ppp4 was predicted from several cDNAs and identified for the first time in a library screening with a PP1 cDNA looking for distinct forms of this enzyme in different tissues. The new protein phosphatase, termed protein phosphatase X or protein phosphatase 4 in the human genome nomenclature, was 41% identical to PP1 and 65% identical to PP2A [78, 79]. Despite its ≈65% amino acid identity to PPP2Cα and PPP2Cβ isoforms and the high similarity with *D. melanogaster* PP4, it was early proposed that mammalian Ppp4 developed distinct cellular roles from PP2A. However, the main subunit of the PP4 complex in *S. cerevisiae* (Pph3) is not essential and was isolated for the first time as a new PP2A-related protein. Whereas double inactivation of the catalytic subunits Pph21 and Pph22 showed a weak defect on spores growth, triple disruption of Pph3 with Pph21 and Pph22 completely prevented growth, indicating that Pph3 provides some PP2A-complementing activity that contributes to the viability of PP2A-deficient cells [44]. According to the overlapping functions with the PP2A orthologues, Pph3 shares a great similarity in amino acid sequence with both Pph21 and Pph22 (82.18% and 82.51%, respectively), implying that this protein could be part of the PP2A holoenzyme. Both mammalian Ppp4 and its counterpart Pph3 contain all the conserved motifs indicative of the PPP family of protein Ser/Thr phosphatases, and they are also closely related to human Ppp6, *D. melanogaster* PPV-6A and *S. cerevisiae* Sit4 [80].

Like most Ser/Thr phosphatases, the substrate specificity of Ppp4 depends on its interaction with its regulatory subunits. In mammals, Ppp4, the catalytic subunit of PP4, is accompanied by two structurally distinct regulatory subunits, termed R1 (105 kDa) and R2 (55 kDa), which do not interact with PP2A. In contrast, the regulatory subunit α4 (39 kDa) dimerises with protein phosphatase catalytic subunits of Ppp4, PP2A and Ppp6 [80]. In *S. cerevisiae*, the open reading frame (ORF) YBL046w, named Psy4, was identified as a putative mammalian R2 orthologue, and Tap42p (two A phosphatase associated protein) as an α4 orthologue [80]. Psy2 (for platinum sensitivity 2) has also been identified as a Pph3 regulatory subunit in *S. cerevisiae*. This protein has two orthologues in humans, named R3A and R3B, which exist in complex with R2-Ppp4 and share an overall sequence similarity with Psy2 [80, 81].

Regarding its molecular functions, PP4 has been implicated in numerous cellular processes. Among them, it is implicated in organelle assembly through its role in centrosome maturation in *D. melanogaster* and *Caenorhabditis elegans* [82, 83], and for spliceosomal assembly via its interaction with the survival of motor neurons (SMNs) complex in mammals [84]. Ppp4 is also involved in several cellular signalling routes including the NF-κB pathway, a key factor in immune and inflammatory responses and tumorigenesis. Ppp4 is thought to dephosphorylate and activate NF-κB in response to cisplatin. This activation is associated with the development of drug resistance against this anticancer treatment. These data suggest that the phosphatase could be the reason behind the increased resistance of some tumor cells to cisplatin [85]. Besides, PP4 is also involved in regulating cell growth both in yeast and mammals in response to nutrients by controlling the TOR pathway. When cells grow on a poor source of nitrogen, Pph3 dissociates from Tap42 and binds to Psy4 to dephosphorylate Gln3 and Ure2. Dephosphorylated Gln3 can move to the nucleus and activate the transcription of genes needed for growth in poor nitrogen sources [86]. In addition, it has been described that Ppp4-R1 complexes decrease the activity of the histone deacetylase HDAC3 by dephosphorylating Ser424, implicating Ppp4 in the regulation of histone acetylation and chromatin remodelling [87]. Finally, budding yeast PP4 controls centromere pairing in meiosis by counteracting Mec1-dependent phosphorylation of Zip1 during the meiotic prophase [88].

The first implication of the PP4 complex in the DDR came from a genome-wide screening to identify proteins required for the repair of DNA lesions generated by UV light. PP4 regulatory subunit Psy2 was identified as a binding partner of Wss1p and Tof1p, which are involved in the response to replication stress. The analysis of genetic interactions between these proteins concluded that Wss1, Tof1 and Psy2 (probably together with the Pph3-Psy4 complex) are implicated in the stabilization of stalled or collapsed replication forks [89]. Over the years, it has become evident that the main role of PP4 in the DDR is to promote cell recovery once the DNA lesion has been repaired. It is well known that DNA damage checkpoint activation requires Rad53 phosphorylation by the DDR-specific kinases Mec1 and Tel1 (yeast homologues of mammalian ATR/ATM), as well as its own auto-phosphorylation. Deactivation of Rad53, a prerequisite to resume cell cycle after DNA repair, is attained through its dephosphorylation by different phosphatases, among them PP4. Pph3 forms a complex with its regulatory subunit Psy2 to dephosphorylate activated Rad53 during recovery from methyl methanesulfonate (MMS)-mediated DNA damage both *in vitro* and *in vivo* [90]. Together with Rad53 deactivation, dephosphorylation of γ-H2AX by PP4 is also necessary for an efficient recovery from the DNA damage checkpoint in *Drosophila, S. cerevisiae* and humans [77, 91, 92]. Besides, depletion of human Ppp4 outcomes in a prolonged checkpoint arrest, in part due to the persistence of MDC1 (mediator of DNA damage checkpoint 1) bound to γ-H2AX at the sites of DNA lesions [92]. These data have been reinforced in budding yeast experiments, demonstrating that the delay in checkpoint recovery manifested in *pph3*Δ cells were alleviated by the expression of a non-phosphorylatable *hta1-S129A* version [91]. Taking into account that there is an additive increase in MMS sensitivity when *PPH3* is deleted in an *hta1-S129A* mutant, it seems that Pph3 function in γ-H2AX dephosphorylation is mechanistically independent of its role in Rad53 deactivation. Confirming this hypothesis, hyper-phosphorylation of γ-H2AX in *psy4*Δ cells does not affect Rad53 dephosphorylation [90]. Surprisingly, PP4 is not only required for DDR deactivation but also for its activation. Pph3 and Psy2 were identified as Mec1-Ddc2 (ATR-ATRIP homologs in humans) regulators in two independent screenings using a *mec1-100* mutant, which is compromised in Rad53 phosphorylation specifically in S-phase arrested cells. Phosphoproteomic analysis revealed that PP4 has the ability to dephosphorylate Ser1991 from Mec1. Interestingly, this function is attained by the physical interaction between Ddc2-Mec1 and the Pph3-Psy2 phosphatase complex at sites of replication fork collapse and DSBs, thus facilitating a coordinated action between the kinase and the phosphatase over many targets in response to replication stress caused by HU treatment [81].

Recently, it has been demonstrated that beyond its functions in checkpoint regulation, PP4 is also involved in DNA repair. Ppp4-R2 complex mediates NHEJ repair of I-SceI-induced DSBs at least partially through regulation of KAP1 (KRAB-associated protein 1) phosphorylation. KAP1 phosphorylation upon DNA damage enhances its binding to chromatin and relaxes it, thereby facilitating DNA repair. Immunoprecipitation assays have revealed that Ppp4-R2 physically associates with KAP1 to dephosphorylate it, since depletion of *PPP4* or *PP4R2* leads to an increase of KAP1 phosphorylation at Ser824 upon camptothecin (CPT) or etoposide treatment [93]. Interestingly, PP4 is also required for recombinational DNA repair by HR. This process requires DNA end resection to allow homology search. The ssDNA generated during resection is immediately protected by the binding of several RPA subunits, including RPA2, which is another target of the human Ppp4-R2 complex in response to replication stress or damage. Depletion of *PP4C* or *PP4R2* results in an increased RPA2 phosphorylation levels, a situation that impedes HR-mediated DSB repair by affecting the loading of the essential factor RAD51 [94]. As for RPA2 dephosphorylation, PP4's role in dephosphorylating H2AX is also essential to stimulate DNA repair. Human phosphatase complex Ppp4-R2-R3B eliminates ATR-mediated γ-H2AX formed with or without exogenous DNA damage generated during DNA replication to enhance a proficient repair of the DNA molecule [92, 95]. This function of PP4 must be evolutionary conserved since yeast Pph3 also regulates basal γ-H2A levels even in the absence of exogenous damage. Importantly, considering that Pph3 does not localize at an engineered DSB, PP4 function in γ-H2A dephosphorylation probably occurs once γ-H2AX has been removed from chromatin [91].

## CDC14: A NEW PLAYER IN THE RESPONSE TO DNA DAMAGE

The cell division cycle 14 (Cdc14) is one of the most studied families within the DUSPs. These proteins are characterized by their ability to dephosphorylate both phosphotyrosine and phosphoserine/phosphothreonine residues in their substrates. The Cdc14 phosphatases family is highly conserved, and orthologs have been described in several organisms. One special feature of this family is its predisposition to dephosphorylate targets of the cyclin-dependent kinase (Cdk). In particular, the Ser/Thr phosphatase Cdc14 was firstly identified in *S. cerevisiae* as an essential cell cycle protein required for Cdk inactivation and mitotic exit [96]. In the budding yeast, the function of this protein is highly regulated by cell cycle-dependent changes in its localization. During interphase, Cdc14 is retained at the nucleolus by interacting with Net1 (also called Cfi1), a subunit of the RENT (Regulator of the nucleolar silencing and telophase exit) complex. As cells enter in early anaphase, Cdc14 spreads first to the nucleus and then to the cytoplasm by the activation of two regulatory networks known as FEAR (Fourteen early anaphase release) and MEN (Mitotic exit network), respectively [97]. In contrast to the high dependence of Cdc14 for exiting mitosis in the budding yeast, the *S. pombe* homologue Cdc14-like phosphatase 1 (Clp1; also known as Flp1) is not essential for accomplishing mitosis [98, 99]. This different requirement of Cdc14/Clp1 between budding and fission yeasts has always been attributed to the distinctive roles of both phosphatases in the execution of the mitotic exit program. However, the fact that a severe depletion of Cdc14 in *S. cerevisiae* by using an auxin-inducible degron does not affect mitosis exit has challenged this point of view, and has reunited both organisms under the common idea of multiple phosphatases cooperating in the removal of Cdk phosphoresidues throughout mitosis [100]. Regarding Flp1 localization during the cell cycle, it localizes at the nucleolus and the spindle pole body (SPB) during G1- and S-phases. However, there are some differences between the budding and the fission yeast in terms of release and activation of the phosphatase. Flp1 release from the nucleolus takes place in metaphase and does not depend on the FEAR pathway [101]. Nevertheless, once released it relocates first to the mitotic spindle and kinetochores and later at the contractile ring, similarly to that in the budding yeast [102]. In vertebrates, three homologous of Cdc14 yeast have been characterized (CDC14A, CDC14B and CDC14C). During interphase, Cdc14A is localized at centrosomes while Cdc14B is mainly nucleolar. As in their yeast counterparts, Cdc14B is also liberated from the nucleolus in anaphase to relocate to the sister chromatids [103]. Little is known about the cellular localization and spatial regulation of Cdc14C. However, taking into account the great similarity in protein sequence with Cdc14B, it is thought that both phosphatases might be genetically redundant.

Regarding Cdc14's molecular functions, multiple studies on different orthologues from yeast to humans have revealed a large number of roles in different cellular processes such as cytokinesis [104, 105], chromosome segregation [106, 107], transcription [106, 108, 109], centrosome duplication [107], ciliogenesis [110] and in resolving linked DNA intermediates [111-113]. Interestingly, it has been demonstrated that Cdc14A and Cdc14B can rescue the lack of Flp1 in fission yeast [114]. In addition, Cdc14A is able to complement the essential function of *S. cerevisiae* Cdc14 in cells lacking the activity of the phosphatase [115]. These evidences suggest that some functional properties of these phosphatases are conserved along different species. In this section we will not discuss the role of Cdc14 in mitotic exit and its regulation during the cell cycle since excellent reviews focused on these topics have been published [107, 116-120].

As mention before, one special feature of the Cdc14 phosphatase family is its predisposition to dephosphorylate targets that have previously been phosphorylated by the Cdk. Since the Cdk controls multiple aspects of the DDR pathway, it is tempting to speculate that the main role of Cdc14 in these processes might be related to its capacity to revert the phosphorylation events imposed by this kinase. In this line, efforts have been focused on understanding the role of this phosphatase family by counteracting Cdk substrates of the DDR. One of the first evidences regarding the role of Cdc14 in DNA damage came from the fission yeast, where Flp1 is translocated from the nucleolus to the nucleus after DNA replication stress induced by HU [121]. Nucleolar release of Flp1 is regulated by the checkpoint kinase Cds1, which is recruited to stalled forks during replication stress. Surprisingly, Flp1 seems to regulate the complete activation of Cds1 through a positive feedback loop that allows a proficient execution of the DNA damage checkpoint in response to replication stress [121]. Corrobo-rating the phosphatase release/activation in response to genotoxic stress, mammalian Cdc14B is also translocated from the nucleolus to the nucleoplasm. Cdc14B liberation promotes the degradation of Plk1 by the ubiquitin ligase APC/C-Cdh1, resulting in the stabilization of the DNA damage activator Claspin and the cell cycle inhibitor Wee1, allowing the proficient G2 checkpoint activation [122]. Together with previous observation demonstrating that Cdc14 is required for Cds1 activation in the fission yeast, it seems that the function of the phosphatase is exclusively related to the activation of the DNA damage checkpoint both in yeast and mammalian cells. Unexpectedly, the publication of two independent studies created controversy about the function of the Cdc14 phosphatases family in the DDR. Using both avian and human somatic cell lines it was demonstrated that neither Cdc14A nor Cdc14B are required for DNA damage checkpoint activation. Indeed, Cdc14A/B knockout cells arrested efficiently in G2-phase without affecting the activation of Chk1 and Chk2 in response to irradiation. However, these cells showed defects in repairing endogenous and exogenous DNA damage, accumulated γ-H2AX foci (as marker for DSBs) and developed hypersensitivity to irradiation [123]. Supporting these results, Cdc14b-deficient mouse embryonic fibroblasts (MEFs) exposed to DNA damage also accumulated endogenous DNA damage and triggered senescence. However, no defects in DNA damage checkpoint activation were observed, indicating that the function of the phosphatase is only restricted to promote efficient DNA repair [124]. The precise mechanism by which Cdc14b is operating in the repair of a DNA lesion is unclear but it seems that Cdc14a is also required for the same process, suggesting that both phosphatases could share at least some substrates and be redundant in terms of function. Supporting this idea, it has been demonstrated that Cdc14B knockout MEFs have defects in repairing DSBs induced by ionizing radiation (IR) but only at late passages when Cdc14A levels are low [125].

One of best-characterized models for the study of the DDR is *S. cerevisiae*. However, the limited evidences regarding a function of Cdc14 in DNA damage response in this organism makes it difficult to understand whether the phosphatase is involved in DNA checkpoint regulation, DNA repair, or both. In this line, it has recently been shown that Cdc14 is transiently released under different sources of DNA damage in the budding yeast [126]. Mass spectrometry data of cells exposed to a DNA lesion revealed multiple targets of the phosphatase in the DDR, suggesting that Cdc14 might have multiple functions in the damage response. Interestingly, it has been found that Cdc14 activity is required for DSB recruitment to the proximities of the SPBs, a vital feature that stimulates DNA repair by HR [126]. Cdc14 stimulates DSB-SPB tethering by dephosphorylating the intranuclear receptor for the γ-tubulin complex Spc110 during the response to DNA damage. Accordingly, both lack of Cdc14 activity and alteration of the steady-state phosphorylation of Spc110 disrupt DSB-SPB interaction and impair DNA repair by HR [126]. These pieces of evidences put forward a new line of Cdc14-dependent DDR regulation by acting over the spatial distribution of its components along the damage response. In this regard, it has been proposed that this layer of regulation is likely to be advantageous for cells to coordinate cell cycle progression with DNA repair, as well as to recruit factors that are required for certain steps during the damage response. This is in line with previous observations demonstrating that unrepairable or slowly repaired DSBs are recruited to the nuclear periphery by interacting with the nuclear pore component Nup84 [127] or the nuclear envelope protein Mps3 [128, 129] in yeast. These mechanisms are believed to collaborate in promoting legitimate DNA repair by HR. It is unclear if this regulation exists in mammals, but the fact that DSB ends are positionally stable in mammalian cells could explain why these cells have a predilection for the NHEJ pathway. Nevertheless, it would be interesting to demonstrate if this new layer of Cdc14-dependent regulation of the DDR is also extended to high eukaryotes.

In addition to the role of Cdc14 in DNA damage checkpoint and DNA repair, it has been proposed that the phosphatase is also required for the resolution of intermediates that are generated during the response to DNA lesions. Interestingly, Cdc14 activity is responsible for the accumulation of the active form of the budding yeast Holliday junction resolvase Yen1 [111-113]. The precise activation of this protein is crucial for the coordination of the DNA repair with chromosome segregation in order to maintain genome stability. These evidences suggest that Cdc14 function in the DDR may be not only restricted to control the damage checkpoint and repair processes, but also the precise resolution of recombination intermediates generated during the response to DNA damage. If Yen1 activation by Cdc14 is an active process that accompanies the repair of a DNA lesion during its damage-dependent release or a passive mechanism operating during the FEAR/MEN activation is an interesting question for the future.

Despite all the information regarding the role of Cdc14 in the DDR, the molecular mechanism by which this phosphatase exerts its function and its regulation during the damage response is still unclear and more effort is required to determine its precise role in the damage response. Remarkably, taking into account the functional redundancy of Cdc14 between different model organisms, it is reasonable to think that the combination of all information can be the answer for the interrogation concerning its role in the DDR.

## INVOLVEMENT OF DDR PHOSPHATASES IN CANCER DEVELOPMENT

During the last years, the repercussion of protein phosphatases in the regulation of the DNA damage response has become clearer. Numerous studies have set the basis for the role of protein dephosphorylation in the regulation of DNA repair at each step of the repair pathway. These observations, together with the fact that protein phosphatases have been implicated in multiple cellular processes, strongly suggest that these enzymes are essential for the maintenance of genome integrity. Supporting this hypothesis, alteration in the expression pattern of several phosphatases and mutations in their sequence have been described in numerous carcinoma cells, highlighting the importance of these enzymes in genome stability. In this regard, protein phosphatases are believed to act either as tumor suppressors or oncogenes [13, 15]. Therefore, they are considered as good candidates for cancer therapy. Accordingly, some experiments have pointed out the use of phosphatases activating drugs to antagonize cancer development and progression. In addition, reactivation of some phosphatases kills cancer cells while spanning normal cells.

One of the first phosphatases directly related to cancer development was PP1. As mention above, PP1 has been involved in the regulation of different cellular pathways, including cell cycle progression and apoptosis, by modulating their main components AKT, APAF-1, AURK, BCL-2, BRCA1, Caspases, CDC25, pRb and p53 [130]. Any disruption in the normal execution of these pathways can lead to the development of cancer so efforts have been focused on understanding the role of PP1 and its regulatory subunits in cell homeostasis. One clear example of its role in tumorigenesis is the fact that PP1 interacts with the breast cancer susceptibility gene *BRCA1* [36, 131, 132]. It is worth mentioning that any alteration in the expression of genes that regulate or interact with BRCA1 can negatively affect to the function of this protein and promote the development of breast cancer. In this line, it has been demonstrated that sporadic breast tumors have variable levels of PP1. Strikingly, some PP1 isoforms are less expressed in breast tumors cells when compared to the normal tissue [132], reflecting the essential role of this phosphatase in cancer development. Supporting these observations, it has recently been postulated that SDS22, a regulatory subunit of PP1, inhibits the growth of breast cancer cells by inducing apoptosis, demonstrating the molecular mechanism by which PP1 negatively regulates the AKT pathway [133]. It is important to note that, due to the role of PP1 in numerous cellular processes, it is difficult to consider this phosphatase as a target for therapy, since the use of drugs that affect the activity of the main catalytic subunit produces a wide range of undesirable toxic side effects. Therefore, the current therapeutic methods are focused on targeting PP1-interacting regulators to alter specific functions of the phosphatase in the DDR. This novel approach was inspired by the realization that most PP1 inhibitors bind to the catalytic subunit through interaction motifs, such as the RVxF docking site. These discoveries have postulated the possibility to modulate a concrete function of PP1 by targeting the RVxF sequence of a particular regulatory element in order to provide a greater selectivity. As a matter of fact, disruption of PPP1C interaction with the regulators GADD34 and HDAC1/6 by treating with the small molecules compounds Salubrinal and trichostaitin A, respectively, has successfully been used in several therapeutic approaches [134].

Together with PP1, PP2A regulates most of the phosphoproteome of the cell. This explains the large number of functions attributed, including modulation of apoptosis, DDR, immune checkpoint signalling and cell proliferation and survival. Interestingly, all these mechanisms are affected during tumorigenesis, mirroring the importance of PP2A in controlling cell homeostasis. Historically, the potential role of PP2A in cancer was postulated when it was reported that okadaic acid, a potent inhibitor of the holoenzyme, had a drastic effect in tumor progression through B-catenin transcription and AKT activation [135]. This evidence was supported by early genetic models of malignant transformation that described the role of PP2A as a tumor suppressor. In addition, several mechanisms that inhibit this phosphatase in cancer have been described [136, 137]. Based on these observations, anticancer therapies involving PP2A have been focused on the activation of the phosphatase in order to prevent cancer development, progression and resistance to other treatments [136, 138, 139]. In this regard, preclinical studies have shown that PP2A reactivation by using FTY720 effectively prevents cancer development, progression and resistance to other treatments [136, 138, 139]. Conversely, inhibition of PP2A to complement chemotherapy and radiation-induced cancer cell death is also an area of active investigation. Direct impairment of PP2A blocks critical defense pathways, rendering cells susceptible to accumulate high levels of DNA damage which ultimately induce apoptosis. On this subject, it has been demonstrated that PP2A's role in cell homeostasis and DNA damage repair has different vulnerabilities in cancer cells, where the inhibition of PP2A coupled with additional DNA damaging strategies may be therapeutically beneficial [139-141].

Alteration of levels or function of PP4 and Cdc14 is also related with malignant transformation and tumorigenesis. PP4 participates in the regulation of microtubule organization, apoptosis, tumor necrosis, immune system signaling and DDR [81, 95, 142-145], pathways involved in cancer development. One of the first evidences that associates the lack of PP4 with tumorigenesis came from *S. cerevisiae*, where *pph3* and *psy2* mutants showed a high sensitivity to cisplatin [146, 147], a platinum-derived drug used in the treatment of solid tumors. Moreover, PP4 was found to be over-expressed in human breast and lung tumors [148], and high levels of this phosphatase are related with aggressive colorectal carcinoma [149]. There are not clear evidences about the involvement of Cdc14 in cancer development. However, due to the important function of the phosphatase in cell cycle and DNA repair, it is reasonable to think that this phosphatase might also be involved in tumor development and progression. Supporting this notion, it has been shown that inactivation of Cdc14 in *S. cerevisiae* leads to chromosome rearrangements and genome instability [150]. Surprisingly, human CDC14A activity is required to control cell adhesion and migration, since the lack of this phosphatase may induce tumor proliferation and metastasis [151]. Indeed, several types of cancer present a down-regulation of CDC14A expression, corroborating the role of this phosphatase in tumorigenesis. Interestingly, overexpression of Cdc14b in mouse cells induces cell transformation due to changes in morphology by disrupting F-actin organization [152]. These data suggest that Cdc14 can act both as an oncogene or a tumor suppressor depending on the context and the cell line. Importantly, it has been postulated that Cdc14b-deficient mouse cells are prone to develop a premature-aging phenotype probably due to the role of the phosphatase in DNA repair [124].

## DDR PHOSPHATASES AND THERAPEUTIC APPROACHES

There are a large number of strategies to modulate phosphatase activity, most of them relying on the use of small molecules that bind to different subunits of the complex to endorse conformational changes that lead to the activation/inhibition of the holoenzyme. To date, several compounds have been identified as inhibitors of the PPP phosphatases. Most of them were originally extracted from natural products generated by organisms with a great biological variability. Probably, the most known inhibitor of protein phosphatases is the okadaic acid, a toxin complex polyether fatty acid produced by dinoflagellates. Its discovery meant a huge breakthrough in the study of Ser/Thr phosphatases function. Multiple studies demonstrated that okadaic acid is a potent inhibitor of PP1, PP2A and PP4 [153]. Similarly, calyculin A, identified from a marine sponge extract, is a potent inhibitor of these phosphatases [154]. Interestingly, inhibition of PP1/PP2A by using okadaic acid or calyculin A has been reported to reduce tumor resistance to radiation or chemotherapy [155], confirming the veracity of these phosphatase inhibitors at the therapeutic level. A variety of cyanophyte strains produce cyclic peptide inhibitors, known as microcystins, which are responsible for the hepatotoxicity of certain species of cyanobacteria. These toxic compounds potently inhibit the activity of PP1, PP2A [156, 157] and PP4 [153]. On the other hand, tautomycin is the only naturally occurring toxin that presents slightly higher affinity for PP1 than PP2A [158, 159]. There are also some antitumor agents able to inhibit phosphatases of the PPP family, like cantharidin or fostriecin. Cantharidin, which is naturally produced by insects, owns inhibitory activity over PP1, PP2A and PP4 [153, 160]. Interestingly, cantharidin has been successfully applied in lung and bladder cancer models, making this new drug a promising alternative for the treatment of these diseases [161]. Regarding fostriecin, identified in *Streptomyces* as tautomycin, is a strong inhibitor of PP2A and PP4, and a weak inhibitor of PP1 [153, 162, 163]. Both of them lead to a G2/M-phase arrest, but their use in oncology is limited due to toxicity or stability problems. Even though phosphatase inhibitors could be idyllic for the cancer treatment, the restricted selectivity of these compounds and the different existing isoforms of some protein phosphatases in the cell, turn the development of specific PPase inhibitors into a very hard task.

While phosphatase inhibition has been the most common therapeutic treatment employed, during the last decade there has been a drastic emergence of a large number of phosphatase activators used as new strategies in cancer therapy. Recently, there have been identified several small molecules that bind to the scaffolding subunit of PP2A to induce the holoenzyme activity, including perphenazine, a tricyclic neuroleptic, SMAP, and a re-engineered version of tricyclic sulfonamide [164-166]. Interestingly, the activation of PP2A can also be tackled by controlling the regulatory subunits of the holoenzyme. The oncoprotein SET is a potent inhibitor of the PP2A holoenzyme, whose levels are dramatically increased in primary chronic lymphocytic leukemia (CLL) and non-Hodgkin lymphoma (NHL) cells. It was reported that COG449, a peptide that binds antagonistically to SET, increased cellular PP2A activity, decreased Mcl-1 expression, and displayed selective cytotoxicity for CLL and NHL cells *in vitro* [167]. These results demonstrate that reactivation of PP2A by modulating its regulatory elements can be considered as a novel treatment in B-cell malignancies. Finally, FTY720, a synthetic sphingosine immunosuppressant that has extensively been used for the treatment of multiple sclerosis, has also been proven to be effective in multiple pre-clinical models of cancer, including leukemia, colon cancer, non-small cell lung cancer, breast cancer, hepatocellular carcinoma and prostate cancer [168]. Importantly, the anti-tumor effect of FTY720 has also been attributed to its ability to disrupt the interaction between SET and PP2A, resulting in an increased PP2A activity and cancer cell death [169]. The same mechanism of action has been proven for ApoE (Apolipoprotein E) and TGI1002. However, the use of these small molecules needs to be further investigated to provide better insights into their mechanism of action and possible side effects.

We have just started to have an idea of the role of protein phosphatases in DNA repair, and much work is required to understand the physiological significance of these enzymes during the repair of a DNA lesion. In this regard, the understanding of the specific functions of each phosphatase in the DDR pathway is vital for the development of new approaches in the treatment of different types of cancer characterized by the alteration of protein phosphatases activity.

## Abbreviations:

CLL: chronic lymphocytic leukemia,
DDR: DNA damage response,
DSB: double strand break,
DUSP: dual-specificity phosphatase,
FEAR: fourteen early anaphase release,
HR: homologous recombination,
HU: hydroxyurea,
MEF: mouse embryonic fibroblasts,
MMS: methyl methanesulfonate
NHEJ: non-homologous endjoining,
NHL: non-Hodgkin lymphoma,
PPP: phosphoprotein phosphatase,
SAC: spindle assemble checkpoint,
SPB: spindle pole body,
ss: single stranded.
